# A155 METASTATIC EPITHELIOID ANGIOSARCOMA AND RECURRENT GASTROINTESTINAL BLEEDING: A CASE REPORT

**DOI:** 10.1093/jcag/gwad061.155

**Published:** 2024-02-14

**Authors:** C Roda, J Chu, A Flynn, R Pai

**Affiliations:** Gastroenterology, The University of British Columbia Faculty of Medicine, Vancouver, BC, Canada; Royal Jubilee Hospital, Victoria, BC, Canada; Royal Jubilee Hospital, Victoria, BC, Canada; Royal Jubilee Hospital, Victoria, BC, Canada

## Abstract

**Background:**

Angiosarcomas are aggressive tumors of the vascular endothelium that represent less than 3% of all sarcomas. Epithelioid angiosarcomas are a distinct subtype, and in exceedingly rare circumstances can involve the gastrointestinal tract leading to significant bleeding.

**Aims:**

Report a case of metastatic epithelioid angiosarcoma as a cause of recurrent gastrointestinal bleeding and the utility of endoscopy in obtaining a diagnosis.

**Methods:**

A retrospective case report.

**Results:**

A 71 year old female with a history of type two diabetes and coronary artery disease presented to a peripheral hospital with a two week history of fatigue, abdominal pain, and intermittent fevers for which she was diagnosed with pyelonephritis. Computed tomography (CT) scan of the abdomen/pelvis was unremarkable on admission. Her stay was complicated by cardiac ischemia prompting the initiation of dual antiplatelet therapy and anticoagulation. After a few days she developed melena and an acute hemoglobin drop requiring red blood cell (RBC) transfusion. Gastroscopy revealed a few superficial erosions in the stomach and duodenum. She went on to develop hypercalcemia prompting a repeat CT abdomen/pelvis which showed multiple lytic bone lesions, innumerable small lung nodules, and areas of hypoattenuation in the liver. Contrast enhanced MRI abdomen was performed to better assess the liver, showing a geographic area of signal abnormality without mass like features. The patient was transferred to a tertiary centre for ongoing work-up and management.

Shortly after arrival, a bone biopsy from a lytic lesion of the iliac crest was taken. Initial histopathology was inconclusive for malignancy. During this time, she had ongoing melena and need for intermittent RBC transfusions. Enteroscopy revealed multiple centrally ulcerated, hemorrhagic, erythematous/purpuric lesions throughout the stomach and proximal small bowel (Figure 1a). A duodenal lesion with fresh clot was clipped and multiple biopsies were taken from the stomach and duodenum. Histopathology from a gastric biopsy confirmed the diagnosis of epithelioid angiosarcoma (Figure 1b). PET scan revealed uptake in bone, lungs, and liver. The patient received 1 cycle of palliative chemoradiation, however this was discontinued due to frailty. Overt bleeding and intermittent RBC transfusions continued. The patient was transferred to full comfort care and died within 4 months of initial presentation to hospital.

**Conclusions:**

Epithelioid angiosarcoma involving the gastrointestinal tract should be considered as a cause for upper gastrointestinal bleeding. Our case highlights the endoscopic characteristics and the importance of endoscopic biopsy in obtaining a histopathologic diagnosis.

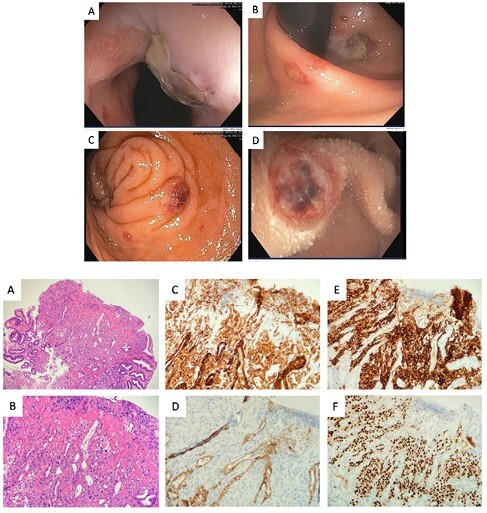

**Figure 1a.** Enteroscopy showing metastatic angiosarcoma to the stomach and small bowel. A. Gastroesophageal junction; B. Cardia and fundus; C. Duodenal bulb; D. Second portion of duodenum.

**Figure 1b.** Metastatic epithelioid angiosarcoma to the stomach. A-B. Low and medium power H&E; C. Cytokeratin AE1/3; D. Ber-EP4; E. CD31; F. ERG.

**Funding Agencies:**

None

